# Medical Student and Resident Dermatology Education in Canada During the COVID-19 Pandemic 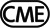


**DOI:** 10.1177/1203475421993783

**Published:** 2021-02-16

**Authors:** Malika A. Ladha, Harvey Lui, Julia Carroll, Philip Doiron, Carly Kirshen, Aaron Wong, Kerri Purdy

**Affiliations:** 1 2129 Division of Dermatology, Department of Medicine, University of Calgary, Alberta, Canada; 2 8166 Department of Dermatology and Skin Science, University of British Columbia, Vancouver, Canada; 3 7938 7989 Division of Dermatology, Department of Medicine, University of Toronto, Canada; 4 6363 Division of Dermatology, Department of Medicine, The Ottawa Hospital, University of Ottawa, Canada; 5 233826 3688 Division of Dermatology, Department of Medicine, Dalhousie University, Halifax, Canada

**Keywords:** COVID-19, medical education, residency, dermatology, telehealth, teledermatology, distance learning, virtual education, virtual care, inverted classroom, undergraduate medical education

## Abstract

The coronavirus disease 2019 (COVID-19) pandemic and subsequent physical distancing recommendations created major gaps in traditional dermatologic undergraduate and postgraduate medical education delivery. Nevertheless, the educational consequences of various public health restrictions have indirectly set aside the inertia, resistance, and risk averse approach to pedagogical change in medicine. In Canada, rapid collaboration and innovation in dermatologic education has led to novel programs including the implementation of a range of internet-facilitated group learning activities and a dramatic expansion of digital telehealth and virtual care. Going forward, three key issues arising from these developments will need to be addressed: the ongoing assessment of these innovations for efficacy; sustaining the momentum and creativity that has been achieved; and, determining which of these activities are worth maintaining when traditional “tried and true” learning activities can be resumed.

The coronavirus disease 2019 (COVID-19) pandemic will profoundly impact undergraduate and postgraduate medical education for the foreseeable future,^1-4^ and it certainly has challenged the current model of dermatology education for residents and medical students.^5-8^ Herein, we highlight educational gaps created by COVID-19, innovative educational initiatives across Canada during the pandemic, and the future opportunities arising from this worldwide health crisis.

## Dermatologic Education Before and Since COVID-19

Post-graduate dermatology education across Canada has traditionally been rooted in supervised direct patient care and separate didactic sessions based on in-person participation at weekly academic half-day sessions, grand rounds, journal clubs and other group activities. The Royal College of Physicians and Surgeons recently mandated the evolution of resident learning and assessment toward a competency by design (CBD) model.^9,10^ This is currently being led by the Specialty Committee in Dermatology for the Royal College of Physicians and Surgeons, with a plan for implementation in 2022.

The Canadian Professors of Dermatology (CPD) also sponsors two well-recognized national education programs through: (1) BoSS (Basics of Skin Science)—an in-person half-day lecture series for senior residents attending the Canadian Dermatology Association (CDA) annual meeting; and, (2) DRIVE (Dermatology Residents Innovation and Vision in Education)—a series of online lectures, practice written exam, and an in-person mock objective structured clinical exam (OSCE) conducted annually for final-year residents preparing for their specialist certification examination. In addition, regional, national, and international conferences have provided important annual opportunities for residents to learn from experts and present their research.

Prior to the COVID-19 pandemic, a few distance education resources were readily available. The CDA produced two seasons of *Dermalogues*, a resident-focused dermatology podcast. Other virtual and accredited programs for the broader dermatology community include the online CDA journal club, the Journal of Cutaneous Medicine and Surgery (JCMS) continuing medical education (CME) program consisting of self-assessment modules, and JCMS author podcasts.

COVID-19 has challenged the traditional delivery of postgraduate dermatology education primarily because of physical distancing measures mandated by public health authorities. This has dramatically reduced in-person clinical activities and didactic instruction in group settings. Likewise, professional and academic dermatology associations have cancelled their in-person meetings due to bans on large group gatherings and nonessential travel.

Undergraduate dermatology education currently exists in the form of pre-clinical teaching followed by clerkship rotations. In Canada, pre-clinical teaching currently represents approximately 75% of a medical student’s total exposure to dermatology, and its format and content varies across Canadian institutions.^10^ While dermatology teaching is gradually increasing within medical school curricula, standardization of pre-clinical dermatology teaching has been suggested to facilitate comprehensive and consistent exposure.^11^

In response to COVID-19, pre-clinical activities have been transitioned to virtual settings and clerkship rotations across Canada were postponed for almost half a year, with an indefinite suspension of inter-university clinical electives. The intermediate or long-term effects of eliminating clinical encounters with dermatology patients and dermatological preceptors on medical student competency are unknown at this time. In addition, the elimination of inter-provincial electives may impact undergraduate students’ chances of matching to dermatology programs during the annual Canadian Residency Match Service (CaRMS). For the upcoming cycle, CaRMS interviews and socials will be completed entirely through virtual means across the country. At this stage, it is unclear how restrictions on electives or the use of virtual modalities for interviews will impact the ability of residency programs to assess candidates or vice-versa.

## Key Educational Changes During COVID-19

This evolving health crisis has demanded flexibility from learners and educators, rapid innovation in education delivery, and collaboration among various stakeholders including the CDA, CPD, CDA Resident & Fellows Society (CDA-RFS), and program directors (PDs) responsible for post-graduate dermatology training across Canada. The current situation has been a forced opportunity to trial pedagogical changes, many of which were likely inevitable, on an accelerated timeline. It has ultimately spurred increased incorporation of virtual modalities into the continuum of clinical and didactic teaching.

COVID-19 has increased acceptance, uptake, and direct experiences with telehealth and virtual care in dermatology.^12^ During the pandemic, telehealth in one form or another has been almost universal in order to maintain even basic levels of essential dermatologic care. Although the mode and extent have varied greatly among practices across Canada, dermatologists have been connecting with their patients through the exclusive or combined use of telephones, real time video, email, text messaging, and “conventional” store-and-forward telemedicine portals.

Telehealth services have been identified as a possible ongoing core service delivery model for rural and remote communities.^9^ While there are some limitations to its use, telehealth will be an upcoming focus for the CBD transition.^9^ From a teaching perspective, teledermatology has been established as an effective dermatologic education tool for both medical students and dermatology residents,^13^ as well as internal medicine and pediatric learners.^14,15^ During the first months of the pandemic, Oldenburg et al. proposed a 4-step method to optimize teledermatology visits for resident education.^6^ However, further validation of this approach is required. Based on the authors’ experiences, residents across Canada have been incorporated into teledermatology visits at varying rates and to varying degrees over the first half-year of the pandemic.

All postgraduate dermatology programs across the country have transitioned their weekly academic half-day sessions to online formats. The close-knit nature of the dermatology community across Canada has facilitated various educational endeavors on a national level. The CDA, CDA-RFS, CPD and PDs have proactively collaborated to establish novel educational initiatives through virtual modalities to support, encourage, and enhance resident learning (Table 1). The recording and storing of lectures has created an online repository that can be accessed later by current and future resident cohorts on the CDA website (www.dermatology.ca).

**Table 1:** Dermatologic Distance Learning Initiatives Established in Canada During the COVID-19 Pandemic.

Educational activity	Sponsors^a^	Structure	Videotelephony technology platform^b^	Time frame	Target PGY level	Average attendance^c^	Record & store	Average Views^d^	Future plans
National Dermatology Lecture Series	PDs CDA CDA-RFS CPD	Faculty across Canada provided 16 weekly lectures on medical dermatology topics	+	First series: April-June 2020	PGY1-5	65	+	32	Future series
National Procedural Dermatology Lecture Series^e^	CDA-RFS CDA	Faculty across Canada provided weekly 17 lectures on procedural (surgical and aesthetic) dermatology topics	+	First series: May-June 2020	PGY1-5	70	+	15	Future series, including formal collaborations with CSDS
National Junior Resident Virtual Bootcamp^e^	CDA-RFS	Faculty and senior residents across Canada delivered 14 lectures on foundational dermatology topics	+	July-August 2020	PGY1 and PGY2	51	+	7	Re-use for future PGY1 cohorts
National Dermatology Morphology Image Challenge (DERMorphIC)	CPD CDA-RFS	Team- and game-based group learning activity for evaluating acumen in visual literacy	+	TBA	PGY1-5	N/A	+	N/A	TBA
Resident Academic Study Hour (RASH)	CDA-RFS	Faculty across Canada provided 20 weekly topic-based examination preparation sessions in a question and answer format	+	First series: June-November 2020	PGY3-5	60	+	12	Future series

Abbreviations: CDA, Canadian Dermatology Association; CDA-RFS, Canadian Dermatology Association Resident Fellow Society; CPD, Canadian Professors of Dermatology; CSDS, Canadian Society of Dermatologic Surgery; PDs, program directors; PGY, post-graduate year; TBA, to be announced.

^a^Leadership personnel from each sponsoring organization initiated and implemented the educational activity.

^b^Zoom® platform.

^c^Residents per live lecture.

^d^Viewings per recorded lecture on the CDA website (www.deramtology.ca) as of 14 October 2020.

^e^Timeline accelerated by the COVID-19 pandemic.

The increasing acceptance of online platforms over the last few months has also accelerated projects that were already in-progress including the National Procedural Dermatology Lecture Series and National Junior Resident Bootcamp. Some of these initiatives have also concurrently responded to educational needs identified before the pandemic. For example, Worley et al. established a need for increased aesthetic dermatologic training in Canadian residency programs^16^; the National Procedural Dermatology Lecture Series has provided increased access to didactic teaching in this realm.

The COVID-19 pandemic has ultimately necessitated a united national approach to curricular activities. This, in turn, has helped to equalize educational opportunities for residents across the country: residents now have increased and organized access to teaching by clinical experts whom they would otherwise not encounter. By uniting residents from coast to coast in educational pursuits, it has also undoubtedly contributed to increased social cohesion among the nascent future dermatology community.

We recognize that these virtual initiatives do not replace traditional clinical teaching activities but rather are supplemental to them. In addition, there are associated barriers such as time constraints (eg, coordinating synchronous teaching sessions across six time zones), limited infrastructure, and “Zoom fatigue,” a recently reported phenomenon of mental exhaustion associated with prolonged and repeated participation on video platforms.^17,18^ It will also remain to be seen whether residents retain the same information and knowledge as with in-person sessions.

## Future Directions

This global crisis provides an unprecedented opportunity to undertake seminal transformation in dermatologic education at the undergraduate and postgraduate level. A variety of possible avenues in the clinical, didactic, and research arenas is summarized in Table 2.

**Table 2:** Future Opportunities for Undergraduate and Postgraduate Dermatology Education.

Setting	Undergraduate	Postgraduate
Clinical exposure	Inclusion of medical students on dermatology electives in telehealth and virtual care for increased clinical exposure	Standardization of institutional policies and protocols for resident-led telehealth and virtual care visits
Didactic teaching	Standardization and harmonization of pre-clinical dermatology curricula across Canada through a national repository of virtual lectures Inverted classroom model Case-based model	Inverted classroom model Separate tracks for junior and senior residents National patient case rounds-based learning Curation and distribution of procedural videos and simulations National online resource database
Research	Potential research areas: Clinical—cutaneous manifestations of COVID-19 Health delivery—evaluation of institutional telehealth and virtual care triage protocols; patient satisfaction with telehealth and virtual care visits; assessment of privacy protection across various modalities Medical education—impact of COVID-19 on current education models; evaluation of telehealth protocols in resident learning Wellness—evaluation of how COVID-19 has impacted burnout rates of dermatologists; assessment of Royal College examination changes on final-year resident wellness Creation of a national virtual research day in which residency programs nominate their top resident research projects for presentation to a national audience Promotion of existing national structures to support Canada-wide research collaboration in collaboration with the Canadian Dermatology Foundation, patient groups, and industry	
Conferences	Use of virtual platforms to harness expert teaching available at in-person conferences Use of online modalities to regularly present research in poster and oral formats Virtual networking sessions with interactive components	

Some components of telehealth will become a permanent pillar of dermatologic care and resident education as we move forward. This would benefit from ongoing evaluation in its effectiveness for patient care and as a teaching tool. It may also provide fruitful and much needed learning opportunities for medical students, all of whom need even a basic level of clinical dermatology exposure. Loh et al. suggests an integrative approach in which residents lead virtual encounters while medical students observe.^7^ With ongoing restrictions on medical school electives, telehealth may be an avenue for postgraduate dermatology residency programs to assess future CaRMS applicants.

E-learning tools have been established as an effective component of resident education in other specialties, including dermatopathology and surgical specialties, and other healthcare domains.^19-22^ While initially spurred by the pandemic, the authors strongly believe that virtual learning will become a permanent instrument of our specialty’s educational armamentarium.

As we develop new programs for residents, consideration should be given to other models such as interactive case-based methods or an inverted classroom. In the latter, individual self-directed learning occurs prior to the classroom instruction in the form of pre-recorded videos, facilitating increased time for application during subsequent case-based discussions or simulations. A recent study demonstrated that a video-based, inverted classroom surgical curriculum is effective in teaching surgical skills to dermatology residents.^23^

Learner enjoyment with different models must also be considered. For example, while the inverted classroom model has been shown to increase learner autonomy and retention of material, it may also decrease the enjoyment of the learning process compared to the traditional model.^24^ On the flip side, the use of an interactive, computerized case simulation system increased learner satisfaction but did not result in any differences on testing of the content.^25^

Assessment and validation of virtual learning tools is critical. A post-initiative evaluation of educational programs created during the pandemic has provided some insight on effectiveness. However, this type of evaluation is limited: it provides a retrospective and reactionary perspective. Going forward, pre-determined teaching objectives and critical evaluations of virtual educational initiatives should be employed. Primary and secondary outcomes, such as knowledge and skill acquisition and retention, learner satisfaction, and cost of creation and implementation, will have to be identified and assessed.

The current situation may be an opportunity to standardize undergraduate dermatologic education across the country. For example, undergraduate dermatology education coordinators could collaborate to develop a national repository of online lectures for medical students. A case-based or inverted classroom model could then be implemented whereby medical students review these lectures prior to sessions organized by their individual institution.

The potential for research projects spurred by COVID-19 in basic science, clinical dermatology, medical education, and wellness realms is broad (Table 2). We strongly encourage members of the discipline to explore these and other research possibilities and to include medical students and residents in these endeavors.

Overall, strong momentum for national virtual education has been initiated for postgraduate learners. With the transition of dermatology to CBD, the authors strongly believe that some distance and virtual education initiatives will become enduring components of resident education. This will necessitate ongoing collaborative efforts for synchronization, sustainability and evaluation. Some initiatives may lose their relevance as we transition back to a “new normal” with less stringent public health guidelines.

## Conclusions

The COVID-19 pandemic has created indefinite gaps in medical student and resident dermatologic education. Nevertheless, this sudden global crisis has accelerated innovation in medical education. As we continue to comply with physical distancing, ongoing implementation of novel techniques, collaboration, and evaluation will be required to maintain rigorous dermatologic education for residents and medical students. These unprecedented and novel changes will likely impact the training of future dermatologists and their physician colleagues.
